# Effects of the perceived temporal distance of events on mental time travel and on its underlying brain circuits

**DOI:** 10.1007/s00221-024-06806-x

**Published:** 2024-03-15

**Authors:** Claudia Casadio, Ivan Patané, Michela Candini, Fausta Lui, Francesca Frassinetti, Francesca Benuzzi

**Affiliations:** 1 Department of Biomedical, Metabolic and Neural Sciences, University of Modena and Reggio Emilia, via Campi 287, Modena, 41125 Italy; 2 Department of Psychology “Renzo Canestrari”, University of Bologna, Bologna, Italy; 3 Istituti Clinici Scientifici Maugeri, Hospital IRCCS, Castel Goffredo, Italy

**Keywords:** Mental time travel, Temporal distance, Functional MRI, Time perception, Spatial representation of time

## Abstract

Mental Time Travel (MTT) allows us to remember past events and imagine future ones. According to previous literature, the Temporal Distance of events affects MTT: our ability to order events worsens for close, compared to far, events. However, those studies established distances a-priori, albeit the way we perceive events’ temporal distance may subjectively differ from their objective distance. Thus, in the current study, we aimed to investigate the effects of Perceived Temporal Distance (PTD) on the MTT ability and the brain areas mediating this process. Thirty-three healthy volunteers took part in an fMRI MTT task. Participants were asked to project themselves into the past, present, or future, and to judge a series of events as relative-past or relative-future, in relation to the adopted time location. Outside the scanner, participants provided PTD estimates for each stimulus of the MTT task. Participants’ performance and functional activity were analyzed as a function of these estimations. At the behavioural level, PTD predicts the modulation of the performance for relative-past and relative-future. Bilateral angular gyrus, retrosplenial cortex, temporo-parietal region and medial, middle and superior frontal gyri mediate the PTD effect. In addition to these areas, the closer the relative-future events are perceived, the higher the involvement of left parahippocampal and lingual gyri and right cerebellum. Thus, perceived proximity of events activates frontal and posterior parietal areas, which therefore might mediate the processing of PTD in the cognitive spatial representation of time. Future proximity also activates cerebellum and medial temporal areas, known to be involved in imaginative and constructive cognitive functions.

**Supplementary Information:**

The online version contains supplementary material available at 10.1007/s00221-024-06806-x.

## Introduction

Mentally travelling in time (MTT) is the cognitive ability to re-experience past events and imagine future scenarios (Tulving 1985). MTT enables humans to disengage from the “here and now” spatio-temporal location and to envision past or future episodes. In addition, travelling towards the past relies on episodic autobiographical memory, while projecting towards the future implies episodic future thinking (Dafni-Merom and Arzy 2020). As proposed in A Theory Of Magnitude (ATOM, Walsh 2003a, b), time, space, numbers and other magnitudes share mapping metrics, and from this theory a spatial representation of time derives. According to ATOM, at the cognitive level, time is represented along a left-to -right line, known as Mental Time Line (MTL; Scozia et al. 2023; Candini et al. 2022; Patané et al. 2016; Ouellet et al. 2010). Thus, we can mentally travel between past and future, represented on the left and right part of such a line, respectively (Bonato et al. 2012; Oliveri et al. 2009). Adopting this view, two main visuo-spatial components of the MTT can be disentangled. The first one is the self-projection component, which is the ability to change the viewpoint from the present time to different moments of subjective time by moving mentally along the MTL (Buckner and Carroll 2007). Thus, self-projection mainly relies on a re-mapping of the egocentric point of view to reconstruct the temporal context (Arzy et al. 2008; Gauthier and van Wassenhove 2016a). Accordingly, being projected in time enables us to change temporal viewpoints relative to a specific event, and this is the self-reference component of the MTT (Arzy, Adi-Japha, et al., 2009a; Arzy et al. 2008, 2009b). As an example, an event which has already happened, such as the Milan Expo in 2015 would be considered as a relative-future event if we projected ourselves to ten years ago (i.e., to 2013). Arzy and colleagues (2008) developed a novel task to investigate these so-called chronometric components of MTT. Participants were instructed to project themselves either to the present, to the past (ten years ago) or to the future (in ten years). They were then asked to judge a series of events as either relative-past or relative-future with respect to the adopted time-location. Participants were slower and less accurate in self-projecting to past and future compared to the present location (Arzy et al. 2008, 2009b, see also Gauthier and van Wassenhove 2016a, b). Moreover, a Temporal Distance (TD) behavioural effect was found: the closer the events to the considered time location, the slower the reaction times (RTs). Interestingly, Arzy and colleagues demonstrated that temporal distance contributes to re-mapping events in the adopted temporal self-location during MTT, since the performance changes according to the temporal distance of events with respect to the adopted time location (Arzy, Adi-Japha, et al., 2009a). Gauthier and van Wassenhove (2016a, b) replicated the temporal distance effect by demonstrating that longer RTs and higher error rate (ER) are required when processing close events. This may result from a complex computation due to the event temporal proximity: the mental representation of an event becomes more detailed and time-consuming when the event is closer in time.

At the neural level, the right temporo-parietal junction (TPJ) may contribute to processing the relationship between the participant’s actual self-location in time and the imagined one during the MTT task (Arzy et al. 2006, 2009b; Blanke and Arzy 2005). Additionally, the right inferior parietal lobule/angular gyrus (IPL/AG; BA 39) and anterior insula participate in judging events as close or far from the participant’s point of view, irrespective of the projection (Gauthier and van Wassenhove 2016b). Furthermore, the distance effect has been investigated in the numerical domain (van Opstal et al. 2008), revealing a specific involvement of parietal regions in numerical quantity processing, calculations and numerical manipulations, and even in implicit processing of quantities (Dehaene et al. 2003).

It is worth noting that none of the previous studies on the temporal distance effect considered the subjective perception of distances from the participants’ point of view. That is, events were a-priori categorised as either close or far (categorical variable), aiming to control for the number of years elapsed from a given event. However, how participants perceived those temporal distances, especially for future personal and non personal events, was not taken into account. Indeed, predictions about future events are expected to vary significantly among individuals, depending on personal background. For example, some participants may perceive the flooding of some Mediterranean islands as imminent in the future due to their knowledge of the extreme effects of climate change, while others may find this event very unlikely to occur. However, the impact of perceived temporal distance (PTD) on performance in temporal tasks, and the specific brain structures mediating this subjective temporal perception have not been previously investigated. Crucially, no previous studies have investigated whether the PTD for relative-past and relative-future events is subtended by the same neurocognitive mechanisms. Assessing whether their neural networks overlap or not can provide insight into the processes involved in the MTT itself.

In the present study, we aim to investigate how the PTD influences the MTT ability and to elucidate the neural correlates of this phenomenon. To achieve this, participants performed an adapted version of the MTT task during a functional Magnetic Resonance Imaging (fMRI) protocol. Then, participants were asked to estimate, in years, the distance of the relative-past and relative-future events employed in the MTT task. To identify the neural basis of the effect of PTD on the MTT ability, we analysed fMRI data as a function of these estimations individually. We expected that the perceived proximity of relative-past and relative-future events in relation to the self-location in time would worsen the performance in the temporal task (Arzy, Adi-Japha, et al., 2009a; Gauthier and van Wassenhove 2016a). Regarding the functional correlates, we predicted the activation of a widespread network comprising medial frontal, retrosplenial and parietal areas (Arzy et al. 2009b; Gauthier and van Wassenhove 2016b; Peer et al. 2015). Moreover, we expected the functional involvement of a temporo-parietal network in the perceived temporal distance processing, irrespective of whether the events were relative-past or relative-future. Specifically, we predicted that this “PTD’s core network” would include IPL/AG and TPJ (Arzy et al. 2009b; Gauthier and van Wassenhove 2016b). In addition to this and specifically for relative-future events, we expected the involvement of brain regions engaged in imaginative and constructive processes (Addis, 2007).

## Materials and methods

### Participants

Thirty-three right-handed (Oldfield 1971) healthy volunteers (20 females and 13 males; mean age 24.9 ± SD 2.5) took part in the experiment. We recruited a sample as homogeneous as possible to reduce interindividual differences in the MTT task related to the succession of events in participants’ life. All participants provided written informed consents, in accordance with the local ethics committee (Comitato Etico dell’Area Vasta Emilia Nord - CE 134/2014/SPER/AOUMO) and the Declaration of Helsinki (2013).

### Procedure

Participants performed an adapted version of the Mental Time Travel task (Casadio et al., 2023; Anelli et al. 2016a), arranged in a jittered single event fMRI protocol. Before entering the scanner for the experimental session, participants were provided with the list of stimuli, so that they could familiarise with the events, in order to avoid any novelty effect during functional data acquisition. The functional session consisted in two acquisition runs of 36 trials each. At the beginning and at the end of each run, 20 s of fixation were introduced to record a baseline for the fMRI signal. A custom-made software developed in our laboratory (http://digilander.libero.it/marco_serafini/stimoli_video/) was used for stimuli presentation via the ESys System (http://www.invivocorp.com) remote display, and for behavioural data collection.

The trial started with a visual warning signal (a blue screen), lasting 500 ms. During the MTT task, the written instructions about the Self-Projection condition appeared on the screen, asking participants to imagine themselves either in the Present (*today*), or in the Past (*ten years ago*), or in the Future (*in ten years*). Instructions were displayed for the entire duration of the trial. 500 ms after the instructions had appeared, stimuli started. Stimuli were auditory two-word descriptions of events, lasting 2000 ms and delivered through MRI compatible headphones. Events were chosen and adapted from a validated list (Supplementary Information Table 1; see also Anelli et al. 2016b). The presentation order of events was pseudo-randomised as a function of the Self-Projection and the Self-Reference condition, resulting in five different sequences of events presented in the task. We chose this approach to control for a possible order effect on the MTT performance. Participants had to classify each event as either “past” -i.e., “occurred before”- or “future” -i.e., “occurred after”- (Self-Reference conditions) with respect to the location in time requested by the Self-Projection instruction. Participants had to respond within a 2000 ms temporal window once the audio was finished. Thus, six experimental conditions were obtained from the combination of Self-Projection (3) and Self-Reference (2):

- Past – relative-past;
- Past – relative-future;
- Present – relative-past;
- Present – relative-future;
- Future – relative-past;
- Future – relative-future.

From now on, we will refer to the Self-Projection conditions using capital letters, i.e., Past, Present and Future, while we will refer to the Self-Reference conditions using the terms “relative-past” and “relative-future”.

**Fig. 1:**
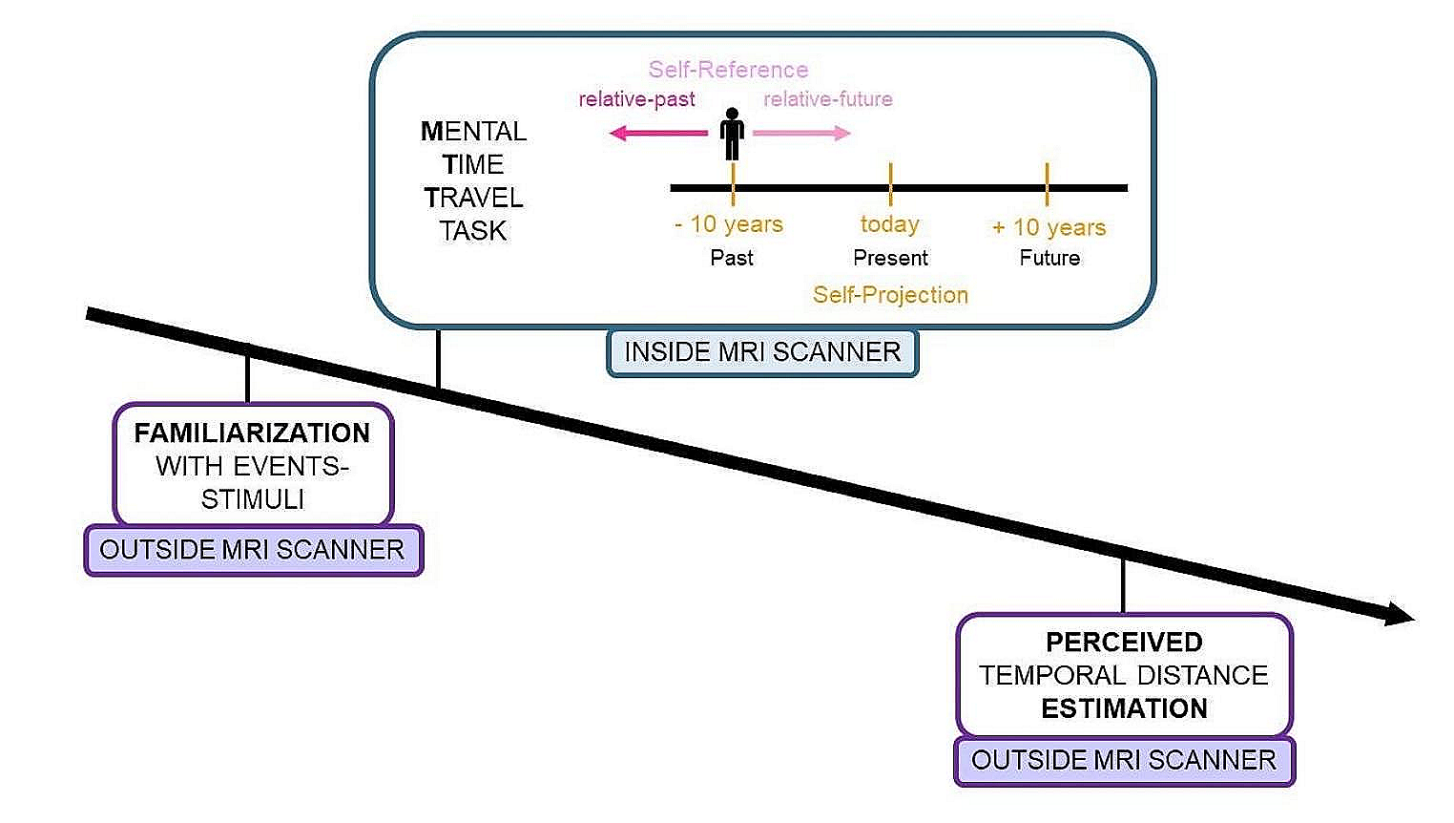
Experimental timeline. At the beginning of the experimental session, outside the MRI scanner, participants familiarised with the stimuli reading the list of the events. Then inside the MRI scanner, participants performed the MTT task, as illustrated in the box. Once the fMRI data collection finished, outside the MRI scanner, participants provided their own personal temporal distance estimates for each event of the task

Participants responded by pressing a two-button keypad either with their index or their middle finger, as quickly and precisely as possible (Fig. 1). In order to avoid motor facilitation due to the spatial representation of time, the responding associations were counterbalanced within participants: half of the participants responded “past” with the index finger and “future” with the middle finger, and the other half used the opposite association. RTs and accuracy were recorded. The inter-stimulus intervals were pseudo-randomised (range 0.5–19.7s) using the make_random_timing.py script from the AFNI package (https://afni.nimh.nih.gov/).

At the end of the functional data collection, participants were interviewed with a questionnaire to verify whether they were aware of the period when each event had / would have occurred. This verification aimed to ensure the accuracy of relative-past/future responses as respect to each individual’s life history. If participants had already experienced an event a-priori categorised as relative-future, or vice versa if they had not yet lived an event a-priori categorised as relative-past, we adjusted the classification to customise the order of events based on the subjects’ experience. To obtain individual evaluations rather than an a-priori categorization of PTD, in a subsequent behavioural session, participants were asked to provide a perceived temporal distance estimation (in years) for each event presented during the MTT task. Negative values were used for relative-past events, while positive values represented relative-future events. Participants provided PTD estimations assuming all three Self-Projection conditions according to the same event order adopted in the MTT task.

### Functional data acquisition

Functional volumes were acquired on a 3T GE Signa Architect system, each of the two runs comprising 320 volumes, each including 46 3-mm-thick slices (TR = 1500 ms, TE = 30 ms, voxel size 3 × 3 × 3 mm, gap 0.3 mm, FOV 24 × 24, matrix 128 × 128). A high-resolution T1-weighted 3D anatomical image (TR = 2184.9 ms, TE = 3 ms, 46 slices, voxel size 1 × 1 × 1 mm) was collected for each participant to allow anatomical localization.

### Data analysis

#### Behavioural data

Pearson’s correlations were conducted between mean absolute values of PTD, calculated across trials, and behavioural performance in each MTT condition, indexed by mean RTs or mean accuracy (percentage of correct answers). Additionally, a series of correlations were computed between mean absolute values of PTD and mean RTs or mean accuracy for each Self-Reference condition, collapsing Self-Projection. To test the causal relation between PTD and behavioural performance we used the individual regression equations method on RTs, as suggested by Lorch and Myers (1990), Bonato et al. (2007) and Pinhas et al. (2012). We ran a single regression analysis for each participant in each MTT condition: mean RTs served as the dependent variable, and the mean absolute values of PTD as the predictor. Then, we performed a series of one tailed t-test against zero on the betas obtained for each participant in each MTT condition.

### Functional data

MatLab R2020a (MathWorks Inc., Natick, MA, USA) and SPM12 software (Wellcome Trust Centre for Neuroimaging, http://www.fil.ion.ucl.ac.uk/spm/) were used for functional data analysis. The following pre-processing steps were run: slice-timing, spatial realignment, normalisation to the MNI template and smoothing with 6 mm full width Gaussian filter. A two-level analysis was implemented. A single-subject statistical analysis was performed applying the General Linear Model (GLM), where the time-series data were modelled as a series of events convolved with a canonical hemodynamic response function. The regressors of interest were as many as the combinations of factors, i.e., the experimental conditions. Motor response, errors, and head-motion parameters (translations and rotations) were entered as nuisance variables. In this single-subject statistical analysis, each individual PTD value was entered as parametric factor matching with its corresponding event of the task (i.e., a single estimated temporal value for each MTT trial). The relationship between brain activity (Blood Oxygenation Level Dependent, BOLD signal) and PTD was modelled with first (linear) polynomial order function. Regressors of interest were as many as the first order functions of each experimental condition (six regressors). The individual contrast images were entered into whole brain analysis at group level and a full-factorial ANOVA with Self-Projection (Past, Present, Future) and Self-Reference (relative-past, relative-future) as factors was conducted for first order relationships. The following contrasts were considered in each analysis (first order relationship):

- Past – relative-past > baseline.
- Past – relative-future > baseline.
- Present – relative-past > baseline.
- Present – relative-future > baseline.
- Future – relative-past > baseline.
- Future – relative-future > baseline.
- Past_projection > baseline.
- Present_projection > baseline.
- Future_projection > baseline.
- relative-past > baseline.
- relative-future > baseline.

Both positive (contrast weight + 1) and negative (contrast weight − 1) relationships were investigated for all the contrasts.

To further explore our dataset, we compared the results from the parametric analysis investigating the PTD effect with those coming from MTT analyses (see Supplementary Information). We masked exclusively the contrast relative-past > baseline in the parametric analysis (i.e., the brain areas which increased their activity for close relative-past events - PTD) with the contrast relative-past > baseline from the MTT analysis (i.e., the brain areas activated when responding to relative-past events). We also masked exclusively the contrast relative-future > baseline in the parametric analysis (i.e., the brain areas which increased their activity for close future events - PTD) with the contrast relative-future > baseline from the MTT analysis (i.e., the brain areas activated when responding to future events). A double statistical threshold was applied to obtain a combined significance, corrected for multiple comparisons, of α < 0.05, as computed by 3dClustSim AFNI routine, using the “-acf” option (https://afni.nimh.nih.gov/pub/dist/doc/program_help/3dClustSim.html). The minimum cluster size for the parametric modulation was 37 voxels.

Given our hypothesis that PTD and RTs are related, we aimed to rule out the possibility that the brain activity possibly associated with the PTD effect may be due to an unspecific time on task effect (i.e., longer RTs), we conducted a parametric analysis on our functional data with RTs (and not PTDs) as parametric factors. If different brain regions were found significantly related to the RTs in the BOLD signal, this would confirm that the activations found as a function of PTDs are specific for the PTD effect, regardless of the time on task. As in previous analyses, a dual statistical threshold was applied to obtain a combined significance level corrected for multiple comparisons (α < 0.05), as computed by the 3dClustSim AFNI routine using the “-acf” option, and the minimum cluster size for the parametric modulation analysis was 69 voxels.

An additional analysis was conducted with the same first and second level parameters of the parametric analysis on PTDs, adding the factor “type of events”, i.e., either personal or public. The following contrasts were considered:

- personal – relative-past > baseline;
- personal – relative-future > baseline;
- public – relative-past > baseline;
- public – relative-future > baseline;
- personal – relative-past > public – relative-past;
- public – relative-past > personal – relative-past;
- personal – relative-future > public – relative-future;
- public – relative-future > personal – relative-future;
- personal_events > public_events;
- public_events > personal_events.

We addressed this issue since a “personal vs public” effect is known to affect the MTT performance: shorter RTs and greater accuracy when responding to personal as compared to public events have been reported (Anelli et al. 2016a, b; Arzy et al. 2008, 2009b).

## Results

### Behavioural results

**Fig. 2:**
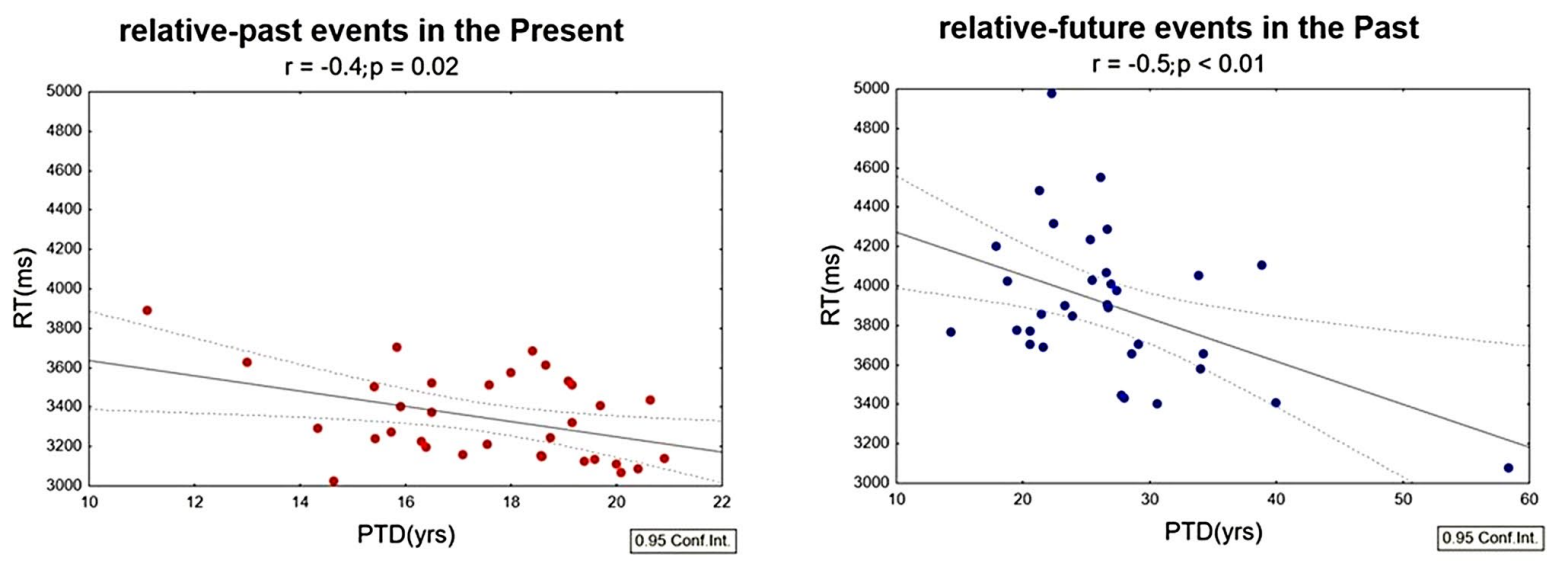
Behavioural results. Mean RTs as a function of mean PTD for relative-past events (absolute value) in the Present self-projection condition (graph on the left) and for relative-future events in the Past self-projection condition (graph on the right)

We found negative correlations between PTDs (absolute value) and RTs both for relative-past events in the Present Self-Projection condition (*r* = -0.4, *p* = 0.02, Fig. 2, left) and for relative-future events in the Past Self-Projection condition (*r* = -0.5, *p* < 0.01, Fig. 2, right). Thus, the closer the events (past and future) the slower the performance. Pearson’s analyses between PTDs and accuracy showed a positive correlation for relative-past events in the Present Self-Projection condition (*r* = 0.5, *p* < 0.01, Supplementary Fig. 1): the closer the past events are to the Present, the less accurate the performance. When considering only the Self-Reference component regardless of the Self-Projection condition, PTDs and accuracy for relative-past events were positively correlated (*r* = 0.5, *p* < 0.01, Supplementary Fig. 2), suggesting that the closer the past events, the less accurate the performance. No other significant correlation was found (for detailed results on the MTT task see Supplementary materials).

The individual regression equations analysis revealed that the averaged negative slopes of the relative-past events in the Past (-0.11 ms; t_32_ = -2.76; *p* < 0.01), relative-future events in the Past (-0.27 ms; t_32_ = -5.01; *p* < 0.001) and of the relative-past events in the Present (-0.10 ms; t_32_ = -1.91; *p* = 0.03) significantly deviated from zero. In addition, also the averaged negative slopes of the overall relative-past events (-0.09 ms; t_32_= -3.11; *p* < 0.01) and overall relative-future events (-0.08 ms; t_32_ = -2.46; *p* < 0.01) were significantly different from zero. This confirms that the closer the events, the slower the performance.

### Functional results

**Fig. 3:**
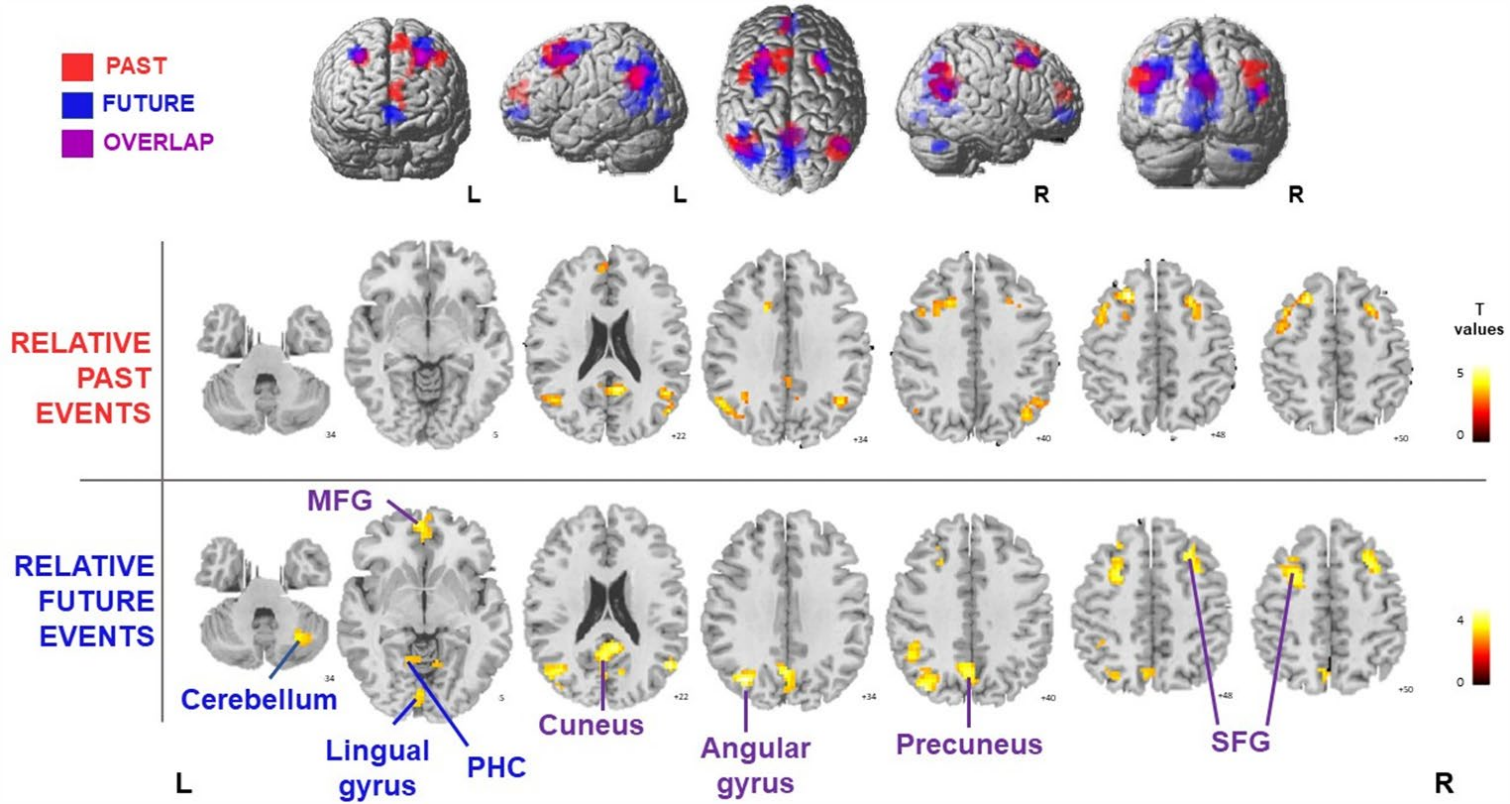
Parametric analysis results. In red the brain areas linearly related with PTDs for relative-past events, in blue the brain areas linearly related with PTDs for relative-future events, purple represents the areas of overlap between the two maps. A double statistical threshold was applied to obtain a combined significance, corrected for multiple comparisons, of α < 0.05 (*p* < 0.001 and cluster size threshold k > 37 voxels). MFG = Middle Frontal Gyrus; SFG = Superior Frontal Gyrus; PHC = Parahippocampal gyrus; L = left; R = right. Functional results are shown on the SPM12 template; color bars represent T-values

The parametric analysis showed significant linear relationships for relative-past and relative-future Self-Reference conditions (i.e., relative-past > baseline; relative-future > baseline). In particular, PTD for relative-past events (expressed in negative values) showed a positive significant relationship (contrast weight + 1) with fMRI signal in several areas, namely, bilaterally in middle and superior medial frontal gyri, cingulate cortex, retrosplenial cortex (precuneus, cuneus), supramarginal and angular (AG) gyri, middle and superior temporal gyri, including temporo-parietal junction (as identified in x = 47.5 ± 0.5, y= -61.5 ± 3.5, z = 20.5 ± 4.5; Geng and Vossel 2013), and in left superior and inferior parietal lobule (IPL), precentral gyrus and pre-supplementary motor area (Table 1; Fig. 3). PTD for relative-future events showed a negative significant relationship (contrast weight − 1) in a strikingly similar pattern of brain areas, with minor differences in peak coordinates and extent (Table 2; Fig. 3). Hence, the closer the PTD for relative-past and relative-future events, regardless of the Self-Projection condition, the more these brain areas are involved. In addition, relative-future events also showed a specific negative linear relationship in left lingual and parahippocampal gyri and right cerebellum (Table 2).

**Table 1:** Results of the linear relationship with PTD for relative-past events

Anatomical regions	BA	Side	Cluster	Voxel level	MNI coordinates		
			K	T	x	y	z
Cingulate Gyrus, Precuneus	23, 30, 31, 7	R	132	5.74	3	-52	26
Middle and Superior Frontal Gyrus, Supplementary Motor Area, Precentral Gyrus	6, 8, 4	L	190	5.68	-24	26	47
Middle and Superior Frontal Gyrus	8	R	45	4.97	24	20	44
Angular Gyrus (AG), Supramarginal Gyrus, Inferior Parietal Lobule (IPL), Middle Temporal Gyrus	39, 40, 22	L	139	4.62	-51	-61	29
Angular Gyrus (AG), Supramarginal Gyrus, Inferior and Superior Temporal Gyrus, Temporo-Parietal Junction (TPJ)	39, 40, 7	R	131	4.54	45	-55	23
Superior Frontal Gyrus, Anterior Cingulate	9, 10	L	43	3.90	-3	59	14

Areas of significant changes in fMRI signal as a function of PTD for relative-past events; BA = Brodmann area; L = left; R = right. A double statistical threshold was applied to obtain a combined significance, corrected for multiple comparisons, of α < 0.05 (*p* < 0.001, k > 37 voxels)

**Table 2:** Results of the linear relationship with PTD for relative-future events

Anatomical regions	BA	Side	Cluster	Voxel level	MNI coordinates		
			K	T	x	y	z
Middle Temporal Gyrus, Angular Gyrus (AG), Precuneus, Superior and Inferior Parietal Lobule (IPL)	22, 39, 7, 30, 19	L	204	4.95	-36	-70	35
Superior and Middle Temporal Gyrus, Angular Gyrus (AG), Temporo-Parietal Junction (TPJ)	22, 39	R	57	4.71	51	-64	23
Precuneus, Posterior Cingulate, Cuneus, Lingual Gyrus, Parahippocampal Gyrus	30, 31, 23,18, 19, 7	L	465	4.58	-6	-52	11
Middle and Superior Frontal Gyrus, Precentral Gyrus	6, 8	L	171	4.26	-27	8	47
Middle and Superior Frontal Gyrus	6, 8	R	53	4.20	30	17	50
Superior Frontal Gyrus, Anterior Cingulate	10, 11, 32	L	60	4.10	-6	56	-4
Cerebellum		R	42	3.88	27	-64	-31
Inferior Parietal Lobule (IPL), Supramarginal Gyrus	40	L	42	3.82	-48	-52	41
Lingual Gyrus	18	L	39	3.62	-6	-82	-7

Areas of significant changes in fMRI signal as a function of PTD for relative-future events; BA = Brodmann area; L = left; R = right. A double statistical threshold was applied to obtain a combined significance, corrected for multiple comparisons, of α < 0.05 (*p* < 0.001, k > 37 voxels)

The masking procedure to identify the brain areas within the MTT network specifically activated as a function of PTD for relative-past events showed the involvement of left superior frontal gyrus (BA 9, 10), right posterior cingulate (BA 23), precuneus (BA 31), bilateral inferior parietal lobule, comprising angular and supramarginal gyri (BA 39, 40), bilateral middle and superior frontal gyrus (BA 6, 8). The same masking procedure for relative-future events revealed the involvement of left medial frontal gyrus (BA 10, 11), anterior cingulate gyrus, angular gyrus (BA 39), middle temporal gyrus (BA 22), precuneus (BA 31), and right superior temporal gyrus (BA 22) and superior frontal gyrus (BA 8). Overall, this explorative analysis seems to highlight a network somewhat similar to the one already revealed from the parametric analysis, further confirming the involvement of these areas in computing the PTD, even when ruling out the MTT related brain activity.

Results of the parametric analysis with RTs as factor revealed significant positive linear relationships between RTs and activity in the left supplementary motor area and bilateral insular cortices both for relative-past and relative-future events (see Supplementary Fig. 3 and Supplementary Tables 2 and 3). Notably, these regions differed from those found in the PTD parametric analysis, allowing us to infer that only these brain regions reflect time on task, whereas the brain activations described in the previous analysis are specifically associated with the PTD effect.

When exploring the potential effect of personal vs. public events, by adding the “type of event” as a factor in the parametric analysis, we did not find any significant results. This suggests that the type of event (personal vs. public) did not explain the brain activity when considering the PTD.

## Discussion

In the present study we investigated how the perceived temporal distance (PTD) of events can affect the Mental Time Travel (MTT) ability and the subtending neural activations. We systematically compared the PTD effect for relative-past and relative-future events in a continuous manner, based on participants’ estimated PTDs, and we analysed the BOLD signal changes as a function of these estimates. Our results showed that reaction time (RTs) and accuracy are linearly related to PTDs for relative-past events in the Present and for relative-future events in the Past. This finding suggests that, even without explicit instructions, participants implicitly represent the temporal distance of events while performing the task. It is noteworthy that the relative-past events in the Present and the relative-future events in the Past can represent the same “segment” of the putative Mental Time Line (MTL). Within this segment, the spatial representation of Past influences event processing, including the PTD effect on RTs, based on the self-location adopted in time (resulting in a similar effect for relative-past events in the Present and relative-future events in the Past). Moreover, PTDs correlate with the accuracy for relative-past events in the Present and the accuracy for the relative-past Self-Reference, regardless of the Self-Projection condition. These results suggest that the PTD effect we observed is stronger for the past: the closer the past events, the slower and less accurate the performance. The regression analyses revealed a distance effect for PTDs in relative-past and relative-future events in the Past, as well as in relative-past events in the Present. This effect persisted for both relative-past and relative-future events when collapsing the Self-Projection conditions. Overall, these findings indicate that a decrease in PTDs results in an increase in RTs, i.e., the closer the events, the slower the reaction time, suggesting a causal linear effect of PTDs on RTs. In previous literature, the distance effect is usually taken as evidence for a continuous representation of magnitudes (Pinhas et al. 2012), which in our case refers to the representation of PTDs on the MTL. Considering previous studies which focused on a-priori dichotomous categorization to study the effect of temporal distance in MTT, our contribution to the MTT literature is especially valuable. Indeed, in the current experiment, PTDs provided by participants did not reproduce a dichotomic evaluation of distances but showed a continuous distribution. Notably, we suggest that these PTDs, rather than the a-priori dichotomic categorization, represent a better predictor of the MTT performance.

It could be pointed out that we did not find the same temporal distance effect for the Future as in previous works (Arzy, Adi-Japha, et al., 2009a; Gauthier and van Wassenhove 2016a, b). This difference could be ascribed to methodological differences between previous studies and the current one. In those earlier works the authors provided participants with the exact date (in terms of years) of the relative-future events that participants had to retrieve when executing the task. In our task, instead, participants had to implicitly estimate the date of each future event which was not a-priori established. If the exact date for relative-past events is already stored in the long term memory and the order of such events are well established on MTL, this is not the case for relative-future events. This difference is crucial and could have masked the perceived temporal distance effect related to the Future, suggesting dedicated mechanisms involved in future MTT. Indeed, to estimate PTDs of future events, cognitive functions, such as anticipation, simulation, mental imagery as well as scene construction processes, are involved. Hence, the heterogeneity of these processes may have prevented the emergence of a future-related temporal distance effect. Anyhow, future research is needed to verify this intriguing hypothesis.

From a neurocognitive perspective, perceiving events as closer results in longer RTs during the MTT task, owing to the activation of more complex representations, i.e., representations rich in episodic and semantic details, which require longer time to be recalled or imagined. Such complexity level decreases as the chronological distance from the present increases (D’Argembeau and Van der Linden 2004). These detailed cognitive representations are tied to considerable brain activity, predominantly in the medial temporal lobe. The hippocampus retrieves and integrates an array of memory details, including sensations, emotions, spatial and contextual details (D’Argembeau, 2020), as well as their temporal order (Gauthier et al. 2020). In particular, the anterior and posterior regions of the hippocampus display different involvement depending upon the detail level, following a gradient from coarse (anterior) to fine details (posterior). In addition, the hippocampus combines segmented information from primary sensory regions and builds event models in the posteromedial cortex (precuneus and posterior cingulate/ retrosplenial cortices) and angular gyrus (D’Argembeau, 2020). Finally, it works alongside parietal areas, particularly inferior parietal cortices, contributing to the vividness of memory recall and allocating attention during this process (Ciaramelli et al., 2010; Cabeza et al. 2008). The complexity of representations is also related to their spatial, temporal or interpersonal distance. According to the Construal Level Theory by Trope and Liberman (2003), closer objects, including events, are represented more concretely and through more detailed “low-level construals”, including specific knowledge, as well as contextual features. This contrasts with more distant events, which are represented more abstractly. From this perspective, the concrete representations tied to proximate events are associated with heightened activity in the anterior and dorsal regions of the medial prefrontal cortex.

The PTD effect here reported is similar to the distance effect observed in the numerical domain (Moyer and Landauer 1967), besides in other temporal tasks (Bonato et al. 2016). The existence of a common mechanism for quantities and distances estimation, be they spatial, temporal, or numerical in nature, has been already suggested, and it implies that the closer an item is to a certain point of reference, the longer it takes to determine the correct answer (for a review see Bonato et al. 2012). In the temporal domain, Bonato and colleagues (2016) observed that right brain damaged patients with left neglect were also impaired in ordering events in time: they exhibited slower responses to items that occurred before the temporal reference provided in the experimental manipulation. Their finding adds further evidence of a common origin of the spatial effects characterising both the numerical and temporal representations of order. In the numerical domain, this distance effect is typically observed in magnitude judgement tasks, where responses become progressively slower and less accurate as the numerical difference between two numbers decreases. van Dijck and Doricchi (2019) demonstrated an asymmetrical numerical distance effect in right brain damaged patients with left spatial neglect. These patients were abnormally slow only when responding to the closest smaller number as compared to the referential number (i.e., 4 when reference is 5). However, patients with spatial neglect showed a normal SNARC effect (Spatial-Numerical Association of Response Codes, i.e., automatic response association between small/large numbers presented on left/right space, respectively - Dehaene et al. 1993), and performed well in parity judgements (“2 is an odd or an even number?”). In the light of this, van Dijck and Doricchi (2019) proposed that spatial neglect does not affect the spatial coded response selection. Namely, the parity judgments can be solved by activating over-learned representation of numbers on the Mental Number Line. Building on this hypothesis, the perceived temporal distance effect reported in this study for the past could rely on an “over-learned” or “well-stored” representation of temporal orders on the MTL, allowing the PTD effect to emerge. Conversely, the lack of defined knowledge about the future could lead to an “interference” of the envision processes of possible scenarios, affecting (or abolishing) the spatialization of the temporal order of relative-future events and, consequently, the PTD effect in the Future.

To the best of our knowledge, our study represents the first attempt to systematically evaluate the modelling effect of PTDs on the BOLD signal in a continuous manner. We found that the perceived proximity of relative-past and relative-future events is associated with the activation of a shared network, encompassing bilateral angular gyrus, temporal and parietal areas, which include the temporo-parietal junction (TPJ, Geng and Vossel 2013), retrosplenial cortex, middle and superior frontal gyri. Our subsequent parametric analysis incorporating RTs as factors confirmed that the activations observed as a function of PTDs are indeed specific to the PTD effect and not influenced by an unspecified time-on-task effect, as other distinct regions are linearly related to the RTs.

Regardless of the specific task employed, a “core network” mediating both the retrieval of relative-past and the envisioning of relative-future events has been described, comprising brain areas very similar to those reported in our results (Arzy et al. 2009b; Benoit and Schacter 2015; Hassabis et al. 2007). This aligns with the recent proposal by Addis (2020) of a single “simulation system”. Notably, our findings revealed the involvement of these areas regardless of whether the events were judged as “past” or “future”. This supports our prediction of a “PTD core network” activated for the processing of perceived temporal distances, especially involving TPJ, inferior parietal lobule, angular gyrus, frontal and posterior areas. This is consistent with Arzy et al. (2009), who identified TPJ as a key structure for the encoding of the self in both the temporal and in the spatial domain. It also aligns with Parkinson and colleagues (2014), who demonstrated that the representations of egocentric spatial, temporal and social distances converge in right TPJ, which is involved in the self-other distinction and in the mental representation of space and events along the Mental Time Line. In addition Gauthier and van Wassenhove (2016b) found a common representation of distances in right IPL/AG and anterior insula both for the temporal and the spatial domains. Right IPL is identified as a pivotal area for egocentric re-mapping and computation of distances in both domains, as well as for the perception of temporal order. Consistently with these findings, Peer et al. (2015) tested distances (close vs. far) in time, space and personal relationship domains, and demonstrated a common activation in the precuneus, IPL, and medial prefrontal cortex. This activation was explained by the processing of the distance between the self-location and the cued stimulus. This observation aligns with the numerical literature, which highlights the crucial role of the posterior parietal cortex, specifically the intraparietal sulcus and angular gyrus, in numerical representations and manipulations. Notably, the left angular gyrus’ involvement in number processing may be linked to the linguistic foundation of arithmetic computations: as this region is also involved in various visuospatial tasks, including eye and/or attention orienting, mental rotation, and spatial working memory, it was identified as a “common ground” for both numerical and spatial domains (Dehaene et al. 2003). Thus, the posterior parietal cortex could also bind numerical and temporal domains.

Our results showed that the brain areas mediating the perceived temporal distance for relative-past and relative-future events do not overlap entirely. This suggests that some of the involved processes could differ, in agreement both with previous literature and with our predictions. It is worth noting that the brain network associated with PTD partly overlaps the Default Mode Network (DMN), which is known to be involved in the self-referential and internal processing (Buckner and Carroll 2007) and in mental travel, allowing for the change of the mental location of the self and the reorganisation of one’s surroundings (Hayman and Arzy 2021). Notably, two subsystems were identified within the core network of the DMN (Addis et al. 2009). The first is the remembering subsystem, activated only during the retrieval of detailed past events (i.e., hippocampus, parahippocampal gyrus and widespread regions of posterior visual cortex; Gaesser & Addis, 2011; Thakral et al. 2017). The second is the imagining subsystem, active when envisioning future scenarios (i.e., anterior hippocampus and widespread medial prefrontal and parietal regions; Addis et al. 2007, 2009). Furthermore, neuropsychological studies revealed that right-brain damaged patients exhibiting left neglect were slower when responding to relative-future than to relative-past events, owing to their spatial working memory deficit (Anelli et al. 2018). Additionally, patients with lesions in the ventro-medial prefrontal cortex were specifically impaired when projecting themselves towards the future and when judging the future location of events on the MTL (Ciaramelli et al. 2021). Furthermore, it has been reported the case of a patient whose gray matter volume reduction in thalamus, fusiform gyri and cerebellum bilaterally, was related to his retrograde amnesia, but also with his impairment in envisioning future events (De Luca et al. 2018). Interestingly, we found that the perceived proximity of relative-future events engages left parahippocampal and lingual gyri and right cerebellum. This is in line with our hypothesis of an additional involvement of processes, such as attention, visual imagery and cognitive resources, in constructing possible future scenarios, when ordering events on the MTL.

The parahippocampal cortex (PHC) is implicated in autobiographical memory retrieval, prospection, navigation, Theory of Mind and Mental Time Travel (Hayman and Arzy 2021; Spreng et al. 2009). Recent studies suggest that PHC also plays a role in constructing scenarios that are alternative to reality (DiNicola et al. 2023). In this regard, Irish and colleagues (2015) found that the gray matter volume of PHC correlates with the ability to construct spatially coherent scenes, contributing to the processing of spatial and contextual associations. Moreover, Epstein and colleagues (2003) demonstrated that the parahippocampal place area (PPA) enables the computation of the location and the orientation of the self with respect to the internal map. In our results, PHC could mediate the representation of future scenes based on the temporal distance of events, envisioning them in spatial terms relative to the location of the self.

Together with the hippocampal and parahippocampal gyri, the lingual gyrus has been identified as a key region mediating creativity and divergent thinking (Dietrich 2004; Gilbert 2001). Namely, Jung et al. (2010) observed that the thinner the gray matter volume of the lingual gyrus, the lower the scores in divergent thinking tasks, particularly in the ideational fluency (i.e., the quantity of original ideas provided). Expanding on these findings, Zhang et al. (2016) demonstrated that a larger volume of the lingual gyrus is associated with increased creativity, as well as with enhanced cognitive flexibility. Furthermore, Zhang and colleagues (2014) highlighted the involvement of the left lingual gyrus in processing relevant visual imagery during the generation of inventive ideas. Additionally, research by Slotnick and Schacter (2006) revealed that the left lingual gyrus plays a role in spatially specific memory processes, implicitly encoding spatial information related to stimuli positioned on the right portion of the screen. Our results could combine all the presented roles of this brain area in the generative process of envisioning future scenarios, since divergent thinking involves retrieving knowledge from memory to creatively organise mental representation as a new idea (Zhang et al. 2020). We speculate that the left lingual gyrus may engage in spatial memory processes to create original future scenarios (located on the right portion of the Mental Time Line), particularly when an implicit representation of the temporal distance of perceived events is considered, as in the MTT task.

Functional MRI studies revealed increased activations in the cerebellum during the processing and the construction of future events (Addis et al. 2007), during episodic future thinking (Szpunar et al. 2006), and when predicting future action sequences during mentalizing tasks (Van Overwalle et al. 2022). Interestingly, Oliveri and colleagues (2009) found that repetitive Transcranial Magnetic Stimulation (TMS) on the right cerebellum of healthy participants affected their speed in responding to the future tense of action verbs. In addition, these authors proposed a right cerebellar-left motor brain network involved in anticipating future events (Oliveri et al. 2009). This hypothesis was confirmed by neuropsychological evidence from patients with cerebellar lesions, whose ability to predict, anticipate and reconstruct sequences of events was impaired (Leggio and Molinari 2015). Furthermore, the functional connectivity between the cerebellum and the mentalizing network (i.e., angular gyrus, parahippocampal gyrus, lateral occipital cortex, middle temporal gyrus and precuneus) was altered in patients with the behavioural variant of Fronto-Temporal Dementia with deficits in social behaviour (Olivito et al. 2022). This evidence strongly supports the role of the cerebellum in generating alternative scenarios based on internal models or past experiences (Oliveri et al. 2009; Schacter et al. 2007). Additionally, our findings suggest the involvement of the right cerebellum in constructing the future temporal location and anticipating relative-future events in the context of the MTT task, especially in relation to the perception of temporal distances. It is plausible that activation of the right cerebellum may facilitate collaboration among brain areas within the PTD core network. This collaboration could be crucial for generating original future scenarios, involving the lingual gyrus for visual processing and PHC for adopting a different spatial perspective. Nonetheless, future research is needed to test the model we propose here, better exploring the co-activation pattern and the connectivity between these regions.

Our findings assume added significance when considered in the context of the hypothesis by Gauthier and van Wassenhove’s (2016b), who proposed that the brain distinctly represents temporal and spatial egocentric distances, as evidenced by the activation of an extensive network specific for the spatial representation of proximity. This network comprised the precuneus/retrosplenial cortex and superior parietal lobule, inferior parietal sulcus, right superior frontal cortex, pre–supplementary motor area, rostrolateral prefrontal cortex, inferior temporal and parahippocampal cortices and left cerebellum. Our study revealed a strikingly similar neural network, however here this network is involved in processing perceived temporal distances. Using both temporal and spatial tasks, Gauthier and van Wassenhove (2016a, b) instructed participants to memorise various details about the events, therefore their temporal task did not involve a spatial component, which was instead prevalent in our study. It is to be noted that, for the first time, in the current study the projection of the self and the localisation of the events on the MTL were executed “on-line” when participants were tested, whereas temporal locations were provided before executing the tasks in Gauthier and van Wassenhove studies (2016a, 2016b). Consequently, the observed similarities between our findings and the activations identified in Gauthier and van Wassenhove study (2016b) for spatial distance computation may be attributed to the spatialization of time induced by our task, which emphasised the spatial representation of time. To support this hypothesis, we selectively masked our neural activity associated with the general MTT process and found that the temporo-parietal, retrosplenial, temporal medial and frontal areas resisted, suggesting that they are specifically involved in the perceived temporal distance computation.

In conclusion, our study provides insights into the mechanisms underlying the processing of subjectively perceived temporal distances in the MTT task, thereby enhancing the ecological validity of the task. The functional imaging findings show posterior parietal, temporal and frontal areas subtending the PTD effect, a network which appears very similar to the one engaged in spatial distances processing. The behavioural results also reproduce spatio-temporal effects related to the distance of events. Thus, our study supports the hypothesis of a common cognitive representation between space and time, as suggested by the ATOM theory (Walsh 2003b; Bueti and Walsh 2009). According to an alternative yet interesting hypothesis, time and space (along with numbers) might interact with each other only at the functional level of working memory. This is suggested by the observation that all three dimensions - time, space, and numbers - require spatial attention and serial ordering within working memory to accomplish tasks (van Dijck et al. 2013). In the same vein, during the MTT task, working memory may build a spatial representation of time, by guiding attention towards long-term memory representations of space and time, thus facilitating the retrieval of past and the imagination of future events. Future research will be needed to disentangle the hypothesis of the spatial representation of time from the spatialization of temporal processes in the working memory.

### Limitations

Our study provides an initial exploration of the role of perceived temporal distance in Mental Time Travel, although our experimental design prevents us from directly comparing the effects of objective and perceived temporal distances on MTT and their associated neural correlates. Future studies, specifically designed to directly contrast these two types of temporal distances, are necessary to enhance our understanding of their implications in MTT and of time perception.

### Electronic supplementary material

Below is the link to the electronic supplementary material.

Supplementary Material 1

Supplementary Material 2

Supplementary Material 3

Supplementary Material 4

## Data Availability

The datasets generated during and/or analyzed during the current study are available from the corresponding author on reasonable request.
