# Supplementary figures and images for: Effects of the perceived temporal distance of events on mental time travel and on its underlying brain circuits
Source: Exp Brain Res. 2024 Mar 15;242(5):1161–74. doi: 10.1007/s00221-024-06806-x (PMC11078804; doi:10.1007/s00221-024-06806-x)

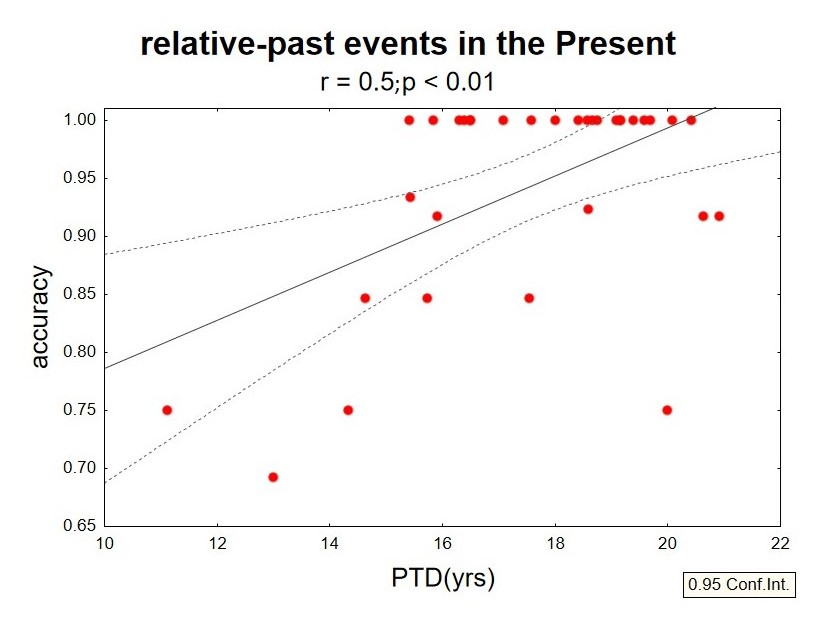

Supplement: Supplementary file 1 — Supplementary Material 1 [file 221_2024_6806_MOESM1_ESM.jpg]

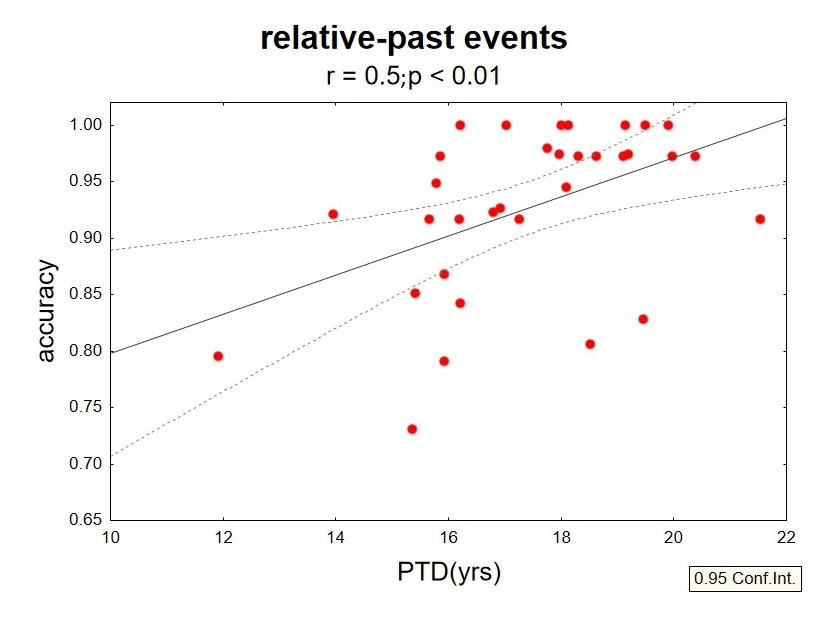

Supplement: Supplementary file 2 — Supplementary Material 2 [file 221_2024_6806_MOESM2_ESM.jpg]

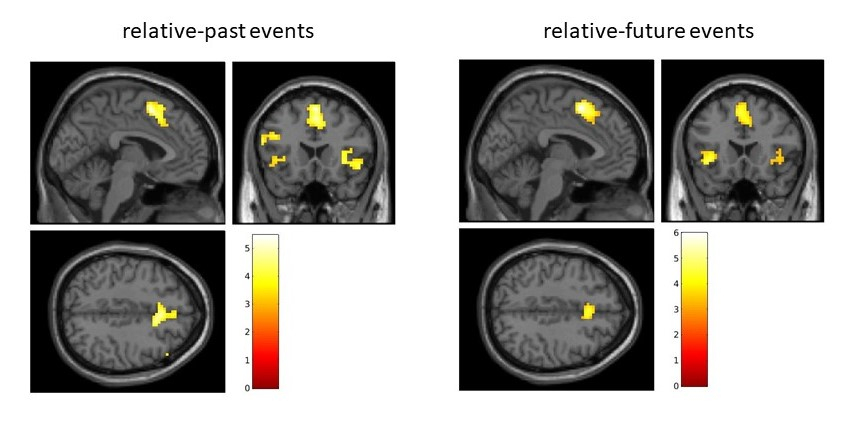

Supplement: Supplementary file 3 — Supplementary Material 3 [file 221_2024_6806_MOESM3_ESM.jpg]
