# Supplementary material for: Effects of the perceived temporal distance of events on mental time travel and on its underlying brain circuits
Source: Exp Brain Res. 2024 Mar 15;242(5):1161–74. doi: 10.1007/s00221-024-06806-x (PMC11078804; doi:10.1007/s00221-024-06806-x)
Supplement: Supplementary file 4 — Supplementary Material 4 [file 221_2024_6806_MOESM4_ESM.docx]

Supplementary Information

**Effects of the Perceived Temporal Distance of events on Mental Time Travel and on its underlying brain circuits.**

Claudia Casadio^1,▲,⋆^, Ivan Patané^2,▲^, Michela Candini^2^, Fausta Lui^1^, Francesca Frassinetti^2,3^ and Francesca Benuzzi^1^

^1^ Department of Biomedical, Metabolic and Neural Sciences, University of Modena and Reggio Emilia, Modena Italy

^2^ Department of Psychology “Renzo Canestrari”, University of Bologna, Bologna, Italy

^3^ Istituti Clinici Scientifici Maugeri, Hospital IRCCS, Castel Goffredo, Italy

^▲^These authors share the first authorship.

^⋆^ corresponding author

e-mail address: claudia.casadio@unimore.it

**List of event stimuli used in the MTT task**

***Supplementary Table 1*** *List of events. Columns indicate the Self-Projection conditions (Past, Present, Future); lines indicate the correct Self-Reference condition (relative-past, relative-future) for each event, divided between personal and public events (Anelli et al. 2016b).*

|  | **PAST** | **PRESENT** | **FUTURE** |
| --- | --- | --- | --- |
| **relative-past- personal** | First best friend | First time at the dentist | Graduate |
|  | First school trip | 10^th^ birthday | 30^th^ birthday |
|  | First time at the sea | First school day | Maturity examination |
|  | Bicycle without wheels | First political vote | First political vote |
|  | 10^th^ birthday | Maturity examination | First salary |
|  | First day of school | Driving licence | Driving licence |
| **relative-future- personal** | 30^th^ birthday | 30^th^ birthday | Living on the moon |
|  | First political vote | First son | Retirement |
|  | First son | Silver wedding | Silver wedding |
|  | Maturity examination | Son marriage | Son marriage |
|  | Leave the hometown | Graduate | Son graduate |
|  | Driving licence | First salary | 50^th^ birthday |
| **relative- past-**  **public** | Princess Diana’s death | Obama’s election | Obama’s election |
|  | Freddy Mercury’s death | Milan Expo | First use of Euro |
|  | Fall of Berlin wall | Notre Dame fire | Gaddafi’s death |
|  | Chernobyl disaster | Charlie Hebdo attack | Pope Francesco’s election |
|  | September 11^th^ | September 11^th^ | Tokyo Olympics |
|  | Man on the moon | Gaddafi’s death | Napolitano’s election |
| **relative-future- public** | Notre Dame’s fire | Peace in middle east | Flying car |
|  | Charlie Hebdo attack | Completely defeat illnesses | World peace |
|  | Milan Expo | Woman president in USA | Completely defeat mafia |
|  | Tokyo Olympics | Completely defeat mafia | Completely defeat illnesses |
|  | Pope Francesco’s election | End of the world | End of the world |
|  | Woman president in USA | Completely defeat world hunger | Completely defeat world hunger |

**Behavioural results: Accuracy**

***
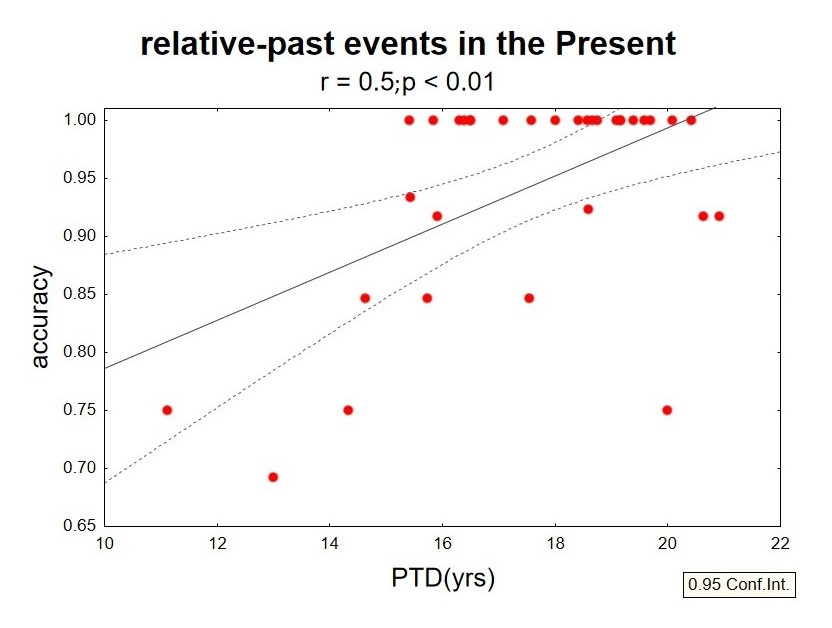
***

***Supplementary Fig. 1*** *Behavioural results. Mean accuracy (percentage of correct answers) as a function of mean PTD for relative-past events (absolute value) in the Present self-projection condition.*

***
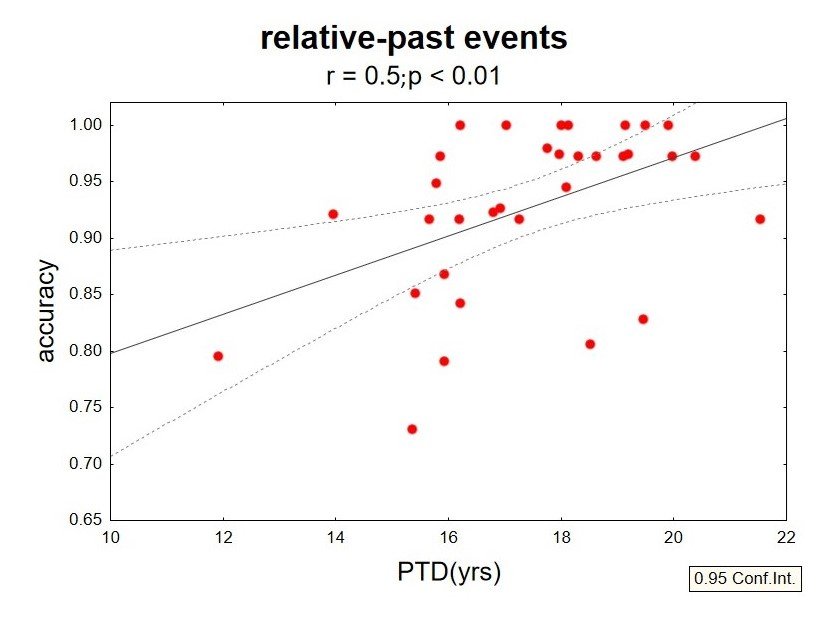
***

***Supplementary Fig. 2*** *Behavioural results. Mean accuracy (percentage of correct answers) as a function of mean PTD for overall relative-past events (absolute value; Self-Reference condition).*

**Functional results: parametric analyses conducted on RTs**


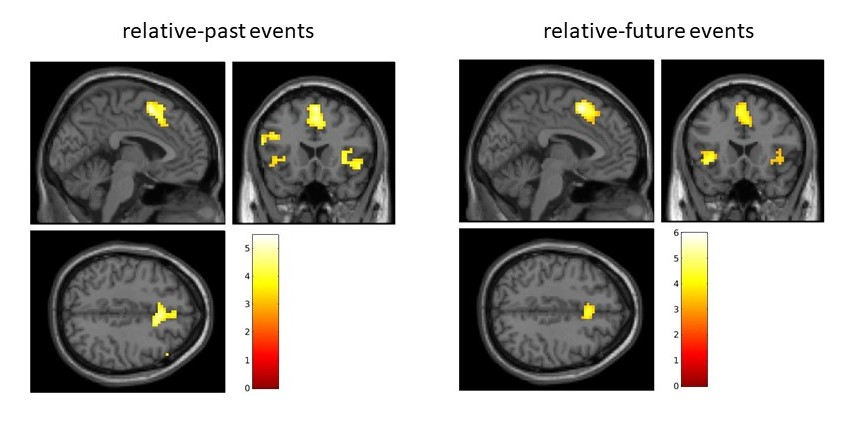


***Supplementary Fig. 3*** *Brain regions whose activity exhibits linear relationships with RTs as a function of Self-Reference conditions. A double statistical threshold was applied to obtain a combined significance, corrected for multiple comparisons, of α < 0.05 (p < 0.001 and k > 69 voxels). Images are in neurological convention (right is right). Color bars represent T-values.*

***Supplementary Table 2*** *Results of the linear relationship with RT for relative-past events*

| Anatomical regions | BA | Side | Cluster | Voxel level | MNI coordinates | | |
| --- | --- | --- | --- | --- | --- | --- | --- |
|  |  |  | **K** | **T** | **x** | **y** | **z** |
| **Supplementary Motor Area, Superior Frontal Gyrus, Cingulate Gyrus** | 6, 8 | L | 323 | 5.45 | 0 | 11 | 53 |
| **Insula, Inferior Frontal Gyrus** | 45, 47 | R | 104 | 4.90 | 33 | 26 | -1 |
| **Insula, Inferior Frontal Gyrus** | 45, 47 | L | 88 | 4.75 | -30 | 26 | -1 |
| **Inferior Frontal Gyrus** | 9 | R | 81 | 4.46 | 39 | 53 | 23 |
| **Inferior Frontal Gyrus** | 9, 10 | L | 142 | 4.32 | -48 | 14 | 26 |

*Areas of significant changes in fMRI signal as a function of RT for relative-past events; BA = Brodmann area; L = left; R = right. A double statistical threshold was applied to obtain a combined significance, corrected for multiple comparisons, of α < 0.05 (p < 0.001, k > 69 voxels)*

***Supplementary Table 3*** *Results of the linear relationship with RT for relative-future events*

| Anatomical regions | BA | Side | Cluster | Voxel level | MNI coordinates | | |
| --- | --- | --- | --- | --- | --- | --- | --- |
|  |  |  | **K** | **T** | **x** | **y** | **z** |
| **Supplementary Motor Area, Superior Frontal Gyrus, Cingulate Gyrus** | 6, 8 | L | 242 | 5.97 | -3 | 8 | 53 |
| **Insula, Inferior Frontal Gyrus** | 45, 47 | R | 76 | 4.93 | 33 | 26 | 2 |
| **Insula, Inferior Frontal Gyrus** | 45, 47 | L | 100 | 4.84 | -39 | 17 | -4 |

*Areas of significant changes in fMRI signal as a function of RT for relative-future events; BA = Brodmann area; L = left; R = right. A double statistical threshold was applied to obtain a combined significance, corrected for multiple comparisons, of α < 0.05 (p < 0.001, k > 69 voxels)*

**Behavioural data analyses and results at MTT task**

Two Separate Repeated-measures ANOVAs were conducted on reaction times (RTs) and accuracy, having Self-Projection (Past, Present, Future) and Self-Reference (relative-past, relative-future) as within-participants factors. Effect size was reported as partial eta squared (η^2^_p_). When significant, interactions were followed-up by Duncan Post-hoc tests. Mean values and standard error means (SEM) were reported for each condition.

Reaction Time

Data showed a significant main effect of Self-Projection (F_2,64_= 76.91; p< 0.0001; η^2^_p_= 0.71) with all the conditions different from each other (mean RT in Past 3866.87 ± 64.1; Present 3365.28 ± 47.4; Future 3625.9 ± 52.2). There was also a significant Self-Projection x Self-Reference interaction (F_2,64_= 6.65; p< 0.01; η^2^_p_= 0.17). Post-hoc analysis showed that performances for relative-future events were significantly faster as compared to relative-past events when participants were projected to the Future (mean 3526.24 ± 50.21 vs 3725.72 ± 68.29 ms; p<0.01).

Accuracy

Data showed a significant main effect of Self-Projection (F_2,64_= 10.63; p< 0.0001; η^2^_p_= 0.25), with significantly lower accuracy in Past Self-Projection (0.88 ± 0.0) as compared to both the other conditions (1; Present 0.92 ± 0.01; Future 0.93 ± 0.01), which did not different from each other . The Self-Reference main effect was also significant (F_1,32_= 8.81; p< 0.01; η^2^_p_= 0.21), with lower accuracy for relative-future as respect to relative-past events (mean 0.89 ± 0.01 vs 0.93 ± 0.01).

**Functional data analyses and results of the MTT task**

Functional data were pre-processed and analysed using MatLab (MathWorks Inc., Natick, MA, USA) and SPM12 softwares (Wellcome Department of Imaging Neuroscience). The following pre-processing steps were used: slice-timing, spatial realignment, normalization to the MNI template and smoothing with a 6 mm full width Gaussian filter. Single-subject statistical analysis was performed using the General Linear Model (GLM), where the time-series data were modelled as a series of events convolved with a canonical hemodynamic response function. Regressors of interest were as many as the combinations of factors, i.e., the experimental conditions. Motor answer, errors and head-motion parameters (translations and rotations) were entered as nuisance variables. Each experimental condition was compared to the baseline and the other conditions and individual contrast images were used for the whole brain random effect analysis. A full-factorial ANOVA with Self-Projection (Past, Present, Future) and Self-Reference (past, future) as factors was conducted on single subject contrast images.

During the MTT task execution a widespread network was activated, comprising right Parahippocampal gyrus and Postcentral Gyrus (BA 1, 2, 3, 30, 36), and bilateral Posterior Parietal Cortex (BA 39, 40), Precuneus (BA 19), Occipital cortex (BA 18, 19), Cerebellum, Basal Ganglia and Inferior, Middle and Superior Frontal gyri (BA 10, 45, 46, 47). For more detailed results, Casadio et al. (in press).
